# Review on Catalytic Biomass Gasification for Hydrogen Production as a Sustainable Energy Form and Social, Technological, Economic, Environmental, and Political Analysis of Catalysts

**DOI:** 10.1021/acsomega.2c01538

**Published:** 2022-07-12

**Authors:** Fikret Muge Alptekin, Melih Soner Celiktas

**Affiliations:** † Solar Energy Institute, Ege University, 35100 Bornova-Izmir, Turkey; ‡ Robert M. Kerr Food and Agricultural Products Center, Oklahoma State University, Stillwater, Oklahoma 74078, United States

## Abstract

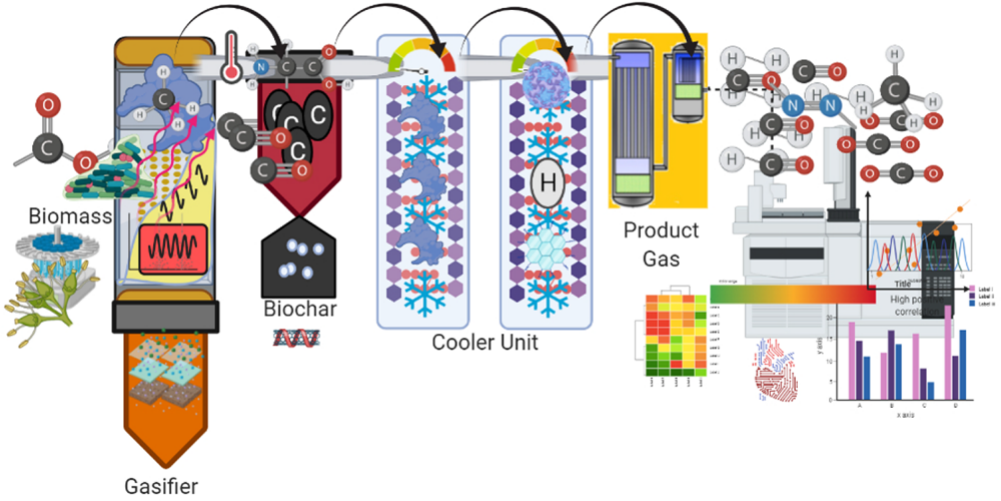

Sustainable energy production is a worldwide concern due to the adverse effects and limited availability of fossil fuels, requiring the development of suitable environmentally friendly alternatives. Hydrogen is considered a sustainable future energy source owing to its unique properties as a clean and nontoxic fuel with high energy yield and abundance. Hydrogen can be produced through renewable and nonrenewable sources where the production method and feedstock used are indicators of whether they are carbon-neutral or not. Biomass is one of the renewable hydrogen sources that is also available in large quantities and can be used in different conversion methods to produce fuel, heat, chemicals, etc. Biomass gasification is a promising technology to generate carbon-neutral hydrogen. However, tar production during this process is the biggest obstacle limiting hydrogen production and commercialization of biomass gasification technology. This review focuses on hydrogen production through catalytic biomass gasification. The effect of different catalysts to enhance hydrogen production is reviewed, and social, technological, economic, environmental, and political (STEEP) analysis of catalysts is carried out to demonstrate challenges in the field and the development of catalysts.

## 1 Introduction

Anthropogenic climate change, which is caused by production and usage of energy, inevitably affects the world by increasing the average global temperature, reducing biodiversity, harmfully impacting nature, and more.^1^ Energy development is crucial to hinder climate change and prevent detrimental effects while sustaining both positive social and economic development.^2^ To decrease the environmental impacts of climate change, a sustainable strategy that includes improved energy efficiency, renewable energy, and sustainable development is necessary.^3^ To achieve a sustainable energy future, renewable energy will have an important role in transitioning to a decarbonized energy system.^4^ However, renewable energy sources such as wind energy and photovoltaics tend to have an intermittent nature, so they need large-scale energy storage for any surplus energy generated.^5−7^ According to Dawood et al., the storage of renewable energy in hydrogen can solve the intermittent generation problem of renewables as hydrogen is storable, transportable, and utilizable.^8^

**Figure 1:**
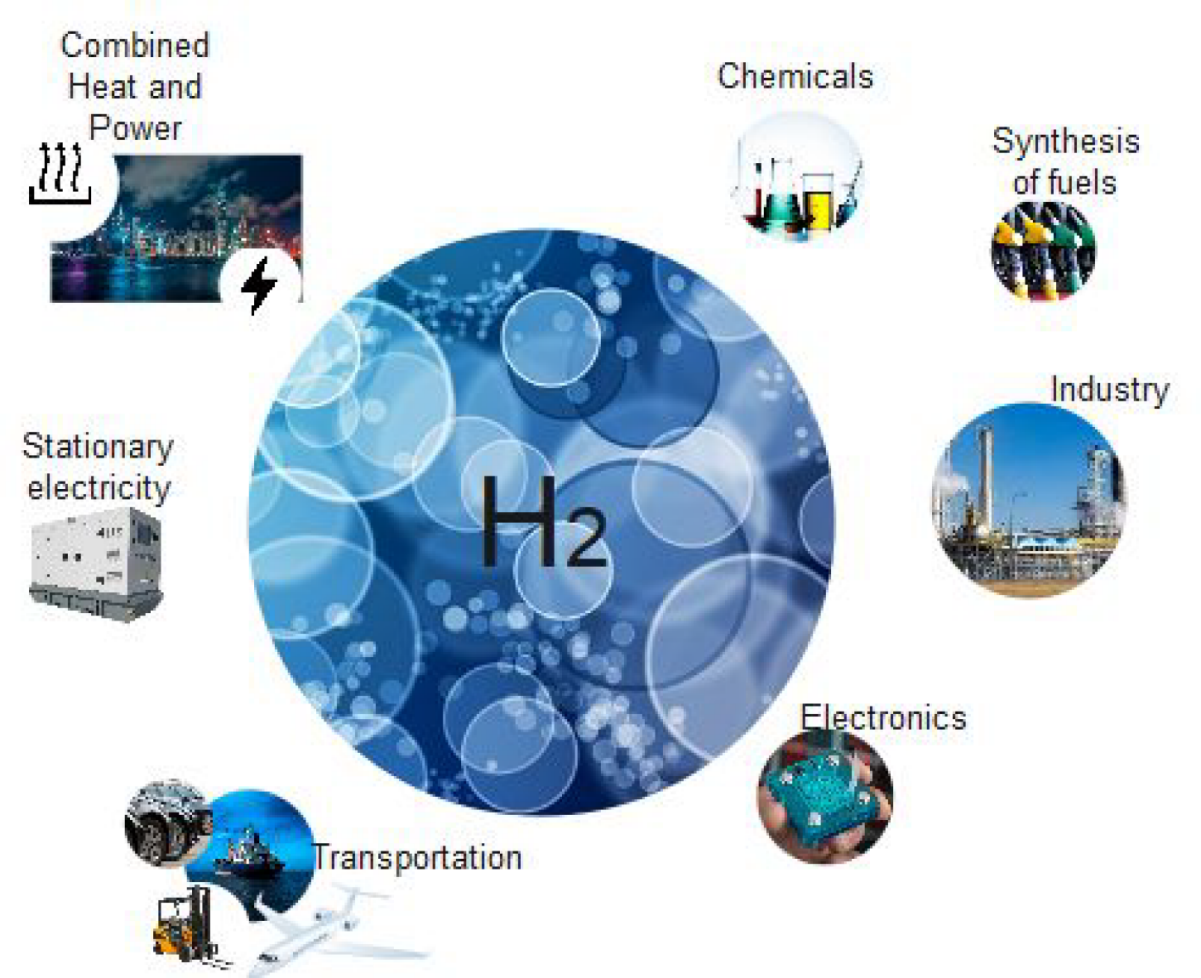
Applications of hydrogen energy (revised with permission from ref (21)).

Due to the accelerating consumption of fossil fuels, increasing energy demand, and environmental problems concerning the use of fossil fuels, hydrogen energy is considered an up and coming pathway to overcome these existing problems.^9^ Similar to electricity, hydrogen is a secondary form of energy that is not a source.^10,11^ Even though hydrogen is not freely available in nature, it is a highly abundant element and can be produced via different methods from fossil fuels and renewable sources.^12−15^ Owing to the fact that it does not emit any toxic products or pollutants during its synthesis from renewable resources and working in fuel cells, hydrogen is considered an environmentally friendly resource.^16,17^ Because of this, hydrogen energy will continue to gain importance in terms of lowering CO_2_ emissions and combating global warming.^18^ In addition to the positive environmental factors, another important factor is the high energy density (122 kJ/g) of hydrogen that allows it to be considered as an alternative fuel.^19,20^ Hydrogen has widespread applications in areas such as stationary electricity, fuel cells for transportation, electronics, heat generation, the chemical industry, synthesis of fuel, and combined heat and power as shown in Figure 1.^21^

Hydrogen is attractive both due to its above-described properties and because it can be generated from both its renewable and nonrenewable resources.^22^ Notably, most hydrogen production is still carried out using fossil fuels in thermochemical processes.^23−25^ As a secondary energy form, hydrogen is produced through three different energy-supply systems: (i) fossil fuels, (ii) nuclear reactors, and (iii) renewable energy resources.^26^ Each supply system has its own advantages and disadvantages with respect to hydrogen production. For example, fossil-fuel-based hydrogen production technologies are mature, although they emit CO_2_ into the atmosphere, which contributes to global warming. In addition, the lifetime of these resources is limited. Alternatively, renewable energy resources have the advantage of being carbon neutral and have the potential to produce hydrogen with appropriate technologies. However, these technologies need to be developed further in terms of efficiency and cost to compete with conventional resources. For example, tar production in biomass gasification is a serious problem that needs to be eliminated to increase H_2_ yield in the product. The cost of producing hydrogen is also an important factor. Although, the efficiencies of the two processes are very close, production costs differ significantly. Producing hydrogen using electrolysis technology costs $10.30/kg, while the cost of producing hydrogen using partial oxidation is $1.48/kg.^27^ In Table 1, the various hydrogen production methods and their resources are listed.

**Table 1:** Various Hydrogen Production Methods^a^

method	resource	description	operation parameters	efficiency	reference
steam reforming	natural gas, methane, and light hydrocarbons (propane, butane, pentane, and light and heavy naphtha)	It includes the catalytic conversion of resources, syngas generation, water–gas shift, and methanation and gas cleaning.	Endothermic reaction	74%–85%	(13, 26, 28, 29)
			Catalytic conversion		
			High temperature, pressures up to 3.5 MPa		
			Steam/carbon ratio = 3.5		
partial oxidation of hydrocarbons	hydrocarbons (methane, heavy oil, and coal)	Syngas production, ammonia synthesis, etc. can be done by the partial oxidation process. It is carried out at relatively high temperatures and elevated pressures.	Exothermic reaction	60%–75%	(13, 27, 30−32)
			–950 °C for the catalytic process		
			–1150–1315 °C for noncatalytic process		
			–5.5–6 MPa of pressure		
pyrolysis	biomass	Thermochemical conversion of biomass to bio-oil, biocrude, and noncondensable gases such as CO_2_, CO, H_2_, and light hydrocarbon gases.	–300–650 °C for catalytic process	35%–50%	(27, 28, 33, 34)
			Lower heating rate and varied feedstock		
gasification	carbonaceous resources include coal, biomass, and petroleum	At high temperatures in the presence of an oxidizing agent, the carbonaceous precursor is converted to syngas that consists of H_2_ and CO.	Endothermic/exothermic reaction	30%–40%	(27, 35−39)
			Temperature range from 500 to 1300 °C		
sub-/supercritical water gasification	biomass	SCWG converts lignocellulosic biomass into gases above 374 °C and 22.1 MPa.	Catalytic SCWG is carried out at 400 °C, while noncatalytic SCWG at 600 °C	-	(40−47)
		Subcritical/near-critical water is carried out at a temperature between 150 °C and 374 °C. Both processes are suitable for wet biomass to convert into H_2_-rich gas products.	Varied residence time, feedstock, and biomass-to-water mass ratio		
plasma arc decomposition	hydrocarbons	Thermal plasma and nonthermal (gliding) plasma is used to decompose hydrocarbons to produce hydrogen. According to a variety of different plasmas and operational conditions, products can show a distribution of results.	In thermal plasma, the temperature ranges from 10 000 K to 100 000 K, there is high current (30 A–30 KA), and low voltage (10–100 V)	-	(48−52)
			In nonthermal plasma, electrons have greater temperatures than the gas components (2200–2500 K)		
biophotolysis	water	Photosynthetic microorganisms (cynobacteria and algae) that enable water splitting are used to reduce protons to hydrogen.	Anaerobic conditions	10%–11%	(27, 53, 54)
			Ferredoxin, reduced ferrodoxin, and reverse hydrogenase are important media		
biological water–gas shift reaction	CO as a carbon source	The process is catalyzed by photoheterotrophic bacteria (*Rhodospirillum rubrum*, *Rubrivivax gelatinosus*) and carried out at ambient temperature and pressure.	Ambient temperature and pressure	100% (near-stoichiometric amount)	(54, 55)
			Dark media		
fermentation	carbohydrate-rich materials (glucose, sucrose, starch, etc.)	It is divided into three types as dark fermentation, photofermentation, and a combination of dark and photofermentation. Organic wastes are decomposed and converted to hydrogen via microorganisms with or without light being present.	The citric acid cycle for photofermentation	–60%–80% for dark fermentation	(27, 56−60)
			Two enzymes, nitrogenase and hydrogenase, are used for the catalytic action in photofermentation	–0.1% for photofermentation	
			In dark fermentation, an acetate-mediated pathway is used for H_2_ production		
solar photovoltaic power	sunlight	Sunlight is converted to electricity by a combination of an electrolyzer and a photovoltaic cell.	Theoretically, a minimum of 1.23 V should be supplied to decompose water to hydrogen	30%	(26, 61−67)
wind power	wind	By using wind energy, water can be electrolyzed, and carbon-neutral hydrogen is generated.	Conventional electrolysis system (alkaline electrolysis or AES) is used	-	(20, 68−70)
hydropower	water	To produce hydrogen, hydroelectric energy is used for power.	Conventional AES system is used	-	(20, 26, 71, 72)
electrolysis	water	Water electrolysis system consisting of movement of electrons. Examples of the technologies are alkaline, polymer membrane, and solid oxide electrolyzers.	Conventional AES system is used	60%–80%	(20, 27, 73−75)
			In an AES, 4.49 kWh/m^3^ of power is required to produce pure hydrogen		

a Abbreviations: Supercritical water gasification (SCWG) and alkaline electrolysis systems (AESs).

It is concluded from Table 1 that steam reforming, electrolysis, and the biological water–gas shift reaction are the best methods with regard to efficiency. Likewise, in every process, the efficiency of biological water shift reactions depends on the bioreactor design, culture media, pH, temperature, etc. Moreover, CO concentration is a limiting parameter for bacterial activity. During consumption of CO as a source, its availability to microorganisms limits the bacterial activity potential. Hence, CO concentration in the feedstock requires optimization. Also, CO toxicity toward bacteria is another inhibiting step of the process. All of these limitations affect the total yield that can be changed from process condition to condition.^76^ Steam reforming is the most widely applied process to produce hydrogen, although it is not an environmentally friendly method since fossil-based resources are used in the process. Instead of steam reforming fossil-based hydrocarbon, hydrogen is produced through a two-stage pyrolysis catalytic steam reforming process. In the first stage, the pyrolysis stage, biomass is thermally degraded to varying hydrocarbons and carbonaceous species. In the second stage, products including hydrocarbons, oxygenated hydrocarbons, and tar are employed in catalytic steam reforming to produce hydrogen-rich syngas.^77^

Considering the environmental concerns and process efficiency, electrolysis is found to be more promising compared to steam reforming of fossil-based resources. Currently, the most commonly used electrolysis technology is alkaline water electrolysis.^78^ In contrast to low efficiency, biomass gasification is considered to be a promising method to obtain hydrogen-rich syngas.^79^

The potential of the different hydrogen production methods seems to possibly change the future energy source. Will this significantly impact how hydrogen production will shape the future? Will it become a green energy form or not? Currently, a great amount of hydrogen is produced via fossil fuels, though this state of affairs can change. In order to eliminate CO_2_ emissions, it is necessary that renewable resource-based hydrogen production methods are adopted.

This study evaluates the catalytic gasification of biomass to generate hydrogen. The scope of the study is to review some of the catalysts that are used in biomass gasification. It includes the catalytic precursors and alternative catalytic materials for enhancing hydrogen production and also tar elimination. Additionally, social, technological, economic, environmental, and political (STEEP) analysis was carried out to demonstrate the challenges of catalysts. The remainder of this paper is organized as follows: biomass gasification (2), catalyst (3), STEEP analysis of catalysts (4), and conclusions and future work (5).

## 2 Biomass Gasification

The term biomass includes both raw materials, such as wood, crops, and agricultural residues, and processed (effluents, food processing residue, and green waste) organic matter. Cellulose, hemicellulose, lignin, lipids, proteins, simple sugars, and starches are the main components of biomass where compositions of the components rigidly depend on whether the feedstock is of plant or animal origin.^80,81^

Biomass has a neutral CO_2_ cycle, meaning it does not emit CO_2_ to the environment when it is processed.^53,56,58,82,83^ Because of this, syngas production from biomass is gaining importance instead of using fossil-fuel-based resources.^84^ There are the two main pathways to produce hydrogen from biomass: thermochemical and biochemical.^85^ Gasification, pyrolysis, and direct combustion are thermochemical processes where biomass can be utilized as feedstock.^86^ Due to the possibilities for polygeneration of other products such as heat, electricity, precious products, or biofuels, biomass gasification has gained more attention than the other thermochemical processes for hydrogen generation.^87,88^

Gasification is one of the thermochemical processes in which fuels or chemicals are obtained by the conversion of carbonaceous materials such as biomass.^89,90^ Biomass gasification is carried out by applying different gasifying agents such as air, steam, oxygen, or a mixture of them.^91−93^ Generally, biomass gasification can be divided into indirect (allothermal)^94^ and direct (autothermal) gasification,^95^ depending on how heat is provided to the systems. Heat that is necessary for gasification reactions is supplied from the outside in indirect gasification, while heat produced directly via partial oxidation of biomass inside the reactor is utilized in direct gasification. Lower heating value (LHV) syngas is obtained in direct gasification by applying air as an oxidizing agent, whereas medium LHV syngas is obtained through indirect gasification.^96^ Due to production of nitrogen free gas, there is no need for any purification. So, indirect gasification has advantages over direct gasification. On the other hand, more energy-efficient utilization is performed via direct gasification thanks to the direct heating of the reactants.^97^ Drying, pyrolysis, reduction, and oxidation are the main steps of biomass gasification.^98^ Very complex reactions including heterogeneous and homogeneous reactions take place during gasification process.^99^

The gasification process is influenced by multiple parameters that have great effects on the end products. The raw material, gasifying agents, operating variables, type of gasifier, and catalysts all have an effect on the amount and heating value of the product gas.^89^ Gasification that is performed at a lower temperature may result in product gas which includes H_2_, CO, CO_2_, methane, and other contaminants, while gasification that is carried out at higher temperatures produces synthetic gas or syngas.^100^ Syngas consists of H_2_, CO, CO_2_, water, light hydrocarbons, and fewer contaminants than product gas.^101−106^

Chen et al. have evaluated the effect of experimental conditions on the production of optimal H_2_ and other gases such as CO, CO_2_, and CH_4_ through the gasification of municipal solid waste (MSW).^107^ In the study, temperature, equivalence ratio (ER), and residence time were chosen as the independent variables in the central composite design to investigate the yield of gases, char, and tar. The optimized H_2_ yield of 41.36 mol % efficiency occurred when experimental conditions were held at 757.65 °C with an ER of 0.241 for 22.36 min. Based on statistical analysis and experimental results, using air as a gasifying agent effectively resulted in both qualitative and quantitative products. For instance, for a steam-to-biomass ratio of 1, mole fractions of CO and H_2_ are 0.52 and 0.15 at 650 °C, while 0.27 and 0.58 mole fraction of CO and H_2_ is reached at 900 °C, respectively.^108^ Singh and Yadav studied steam gasification of mixed food waste at 700 °C.^109^ They employed torrefaction as a pretreatment method to improve the physicochemical properties of the mixed food waste. In the study, the steam-to-biomass ratio was chosen as 1.25, and the steam ratio was held constant at 0.625. Their results showed that syngas production increased with the increasing temperature of torrefaction. Torrefied food waste at 290 °C gave the highest hydrogen yield with 2.15 m^3^/kg.

The gasifying agent is one of the parameters that highly influences the syngas composition, yield, and calorific value. Singh et al. investigated different steam flow rate (from 0.125 to 0.75 mL/min) and temperature (700–900 °C) effects on the syngas yield, syngas composition, and hydrogen yield.^110^ In the study where food waste was used as feedstock, the highest hydrogen yield achieved was 1.23 m^3^/kg at a 0.5 mL/min steam flow rate and 800 °C temperature.

The biomass feedstock and the characteristics of the biomass are important parameters that seriously affect hydrogen production. The chemical constituent of the biomass (cellulose, hemicellulose, and lignin), elemental composition, mineral content of the biomass, amount of volatile matter, moisture content, and physical properties such as particle size, shape, and density all affect gas composition and yield in biomass gasification.^111−113^ According to Tian et al., cellulose and hemicellulose resulted in more CO and CH_4_ contamination, while biomass with a higher percentage of lignin produced more hydrogen.^114^ Moreover, increasing the gasification temperature enhances hydrogen production, while increasing the steam-to-biomass ratio influences the yield of hydrogen in syngas^111^ until the limit of gasification stoichiometry.^115^ Steam methane reforming and dry reforming reactions are raid reactions that occur at temperatures higher than 700 °C. These lead to increased syngas production through improvement of secondary cracking and shift reactions. Higher S/B ratios in the gasification process cause the gasification temperature to decline, leading to poor syngas quality. Above this limit, a decrease in the gasification temperature and product quality is observed.^116^Table 2 shows the effect of biomass type, reactor design, operation conditions, and gasification agent on syngas yield, composition, and hydrogen content. It can be seen from Table 2 that gasification parameters have a serious effect on the gaseous products and their composition.

**Table 2:** Effect of Gasification Parameters on Syngas Yield, Composition, and Hydrogen Content^a^

biomass type	reactor type	catalyst	operation conditions	gasification agent	syngas yield and composition	hydrogen content	ref
switchgrass (SG), pine residue (diameter of ≤2 in. and of ≤6 in.)	bench-scale fluidized bed	catalyst bed material: sand, CaO + sand, Al_2_O_3_, and CaO + Al_2_O_3_	–780 °C and ER ≈ 0.32	air/steam	H_2_ (32.1%), CO (7.5%), CO_2_ (21.8%), CH_4_ (2.5%), C_2_H_2_ (0.01%), C_2_H_4_ (1.9%)	The highest H_2_ of 32.1 vol % with S/B of 2.34 for Pine6 using the CaO + Al_2_O_3_ bed material.	(117)
			steam-to-biomass ratios (S/B) (0.74, 1.23, 1.85, and 2.34)				
cellulose, hemicellulose, lignin, poplar leaf, Chinese cabbage, and orange peel	updraft fixed-bed reactor	no catalyst	varied temperature range (920–1220 °C)	steam	H_2_ (54%), CO (26.2%), CO_2_ (18.8%), CH_4_, C_2_H_4_ (−), and C_2_H_2_ (−) for lignin at 920 °C	The highest H_2_ yields for cellulose, hemicellulose, and lignin were 0.27 N m^3^/kg (1220 °C), 0.30 N m^3^/kg (1220 °C), and 0.88 N m^3^/kg (1020 °C), respectively.	(114)
wood pellets	bench-scale fixed-bed gasifier	no catalyst	high temperature (800–1435 °C)	steam	H_2_ (60%), CO (≈13%), CO_2_ (≈18%), CH_4_ (≈6%), C_2_H_4_ (≈1%), and C_2_H_2_ (≈1%)	The maximum volume percentage of H_2_ was 60% at 917 °C with 9.0 g/min of steam flow rate.	(118)
			steam flow rates (3.9, 4.7, 5.5, 6.8, 9.0, 9.8, 11.1, 15.7, and 17.3 g/min)				
municipal solid waste	tube reactor	no catalyst	different temperatures (700, 800, 900 °C), ERs (0.1, 0.2, 0.3), and residence times (10, 20, 30 min).	air	H_2_ (32 mol %), CO (34.7 mol %), CO_2_ (28.6 mol %), and CH_4_ (4 mol %)	The highest H_2_ yield of 32 mol % was achieved at 900 °C with an ER of 0.25 and 20 min of residence time.	(107)
citrus peel	bench-scale fluidized-bed reactor	no catalyst	different temperature range (700–850 °C)	air-steam	H_2_ (26.5%), CO (≈8%), CO_2_ (20%), N_2_ (≈42%), and CH_4_ (≈3%) at 750 °C and S/B = 1.25 for experimental results	The highest H_2_ yields of 0.65 and 0.69 N m^3^/kg were achieved at 750 °C and S/B = 1.25 for the experimental and simulated results, respectively.	(119)
			(S/B) (0.5–1.25)				
banana peel	fixed-bed gasifier	no catalyst	different steam-to-carbon ratios (S/C) (0, 0.6, 1.4, 4.3, 7.2, 14.5, 21.7, 28.9, and 36.1)	steam	CO_2_ (≈33%), CH_4_ (≈2%), C_2_ (≈2%), CO (≈8%), and H_2_ (≈58%)	The maximum value of 76.1 mL/g of H_2_ yield was achieved at S/C = 21.7 and a temperature of 1023 K.	(120)
algal biomass (*Nannochloropsis* sp)	hydrothermal carbonization (HTC) and a laboratory-scale quartz tube reactor	no catalyst	for HTC (180–220 °C) and reaction time (2, 6, 12 h)	steam	H_2_ (≈46%), CO (≈32%), CO_2_ (≈16%), and CH_4_ (≈6%) of feedstock of HC-180 °C-12 h at 800 °C with S/B ratio of 3	The maximum H_2_ concentration of 48.6% was achieved with HC-220 °C-12 h, whereas further gasification optimization was continued with feedstock of HC-180 °C-12 h due its high ER*E*_total_.	(121)
			gasification temperature (700–900 °C)				
			S/B ratio (1–3)				

a Abbreviations: equivalence ratio (ER), steam-to-biomass ratios (S/B), steam-to-carbon ratios (S/C), hydrothermal carbonization (HTC), total energy recovery efficiency (ER*E*_total_).

Apart from the advantages of biomass gasification, tar formation is a serious problem that faces the adoption of this process.^116,117^ Due to the condensation of tar, equipment becomes blocked, and engines and turbines are damaged, resulting in costly maintenance and gas cleaning.^122−127^ Additionally, tar condensation causes cracking in filter pores and adverse effects on the cold gas efficiency and heating value of the produced syngas. Besides tar formation, pollutants such as NH_3_, H_2_S, HCl, SO_2_, dust, ash, etc. can be found in syngas, which might affect the applications of syngas.^128^ As mentioned before, apart from the quality and quantity of gaseous products, biomass feedstocks, gasifying agents, reactors, and the activity of catalysts also have an enormous effect on tar formation.^129−131^

Complex polycyclic aromatic hydrocarbons (PAHs), oxygen-containing hydrocarbons, and monocyclic hydrocarbons generate multiple condensable organic compounds called tar.^129^ Some of the problems of tar condensation are that it is highly stable, refractory, not easy to crack, and causes coke formation on the surface of catalysts.^132^ Tar is produced through three stages, and tars formed at each stage are classified as primary, secondary, and tertiary. Primary tar formation occurs during the pyrolysis step of gasification. When feeding biomass into a gasifier, pyrolysis first takes place at a low temperature around 200 °C and ends at 500 °C. After 500 °C, primary tar reorganizes into secondary tar that includes lighter noncondensable gases and some heavier molecules. At even higher temperatures, tertiary tar is generated.^133^ Toluene, naphthalene, other one- and two-ring aromatic hydrocarbons, phenolic compounds, heterocyclic compounds, and polyring and cyclic structures form biomass tar.^134^ Both reforming and cracking reactions are recognized for biomass tar cracking as shown in eq 1–7:^135^
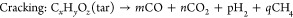
1
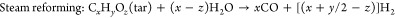
2
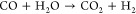
3
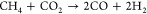
4
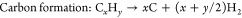
5
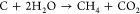
6
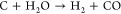
7In order to enhance gas quality^136^ and prevent adverse effects of tar compounds on the gasification system and its components,^137^ effective tar reduction is required. In the next section, a brief description of catalysts, tar reduction methods, and different types of catalysts that are used in hydrogen production is given.

## 3 Catalyst

A catalyst is a material that, by adding a small amount, accelerates the rate of a chemical reaction without undergoing any chemical change itself. Successful catalysts lower the required activation energy for gasification reactions, decrease both the temperature and time for the process, and yield high carbon conversions that are beneficial for the gasification process.^138,139^ Catalysts help to decrease the required temperature for the gasification process and tar production.^140^ Abedi and Dalai examined the steam gasification of oat hull pellets with and without catalysts to produce syngas and reduce tar formation.^141^ The effect of temperature (650–850 °C) and the steam-to-biomass ratio (0.25–0.50) were evaluated in noncatalytic gasification. Higher temperature and increased steam-to-biomass ratio raised fuel gas production, heating value of the gas, and reduced tar condensation. Synthesized Ni/Al_2_O_3_ catalysts with and without Ce were used for catalytic gasification, and all experiments were carried out at 650 °C with a catalyst-to-biomass ratio of 0.5. The lowest tar formation was achieved by 10% Ni loading of the catalysts, while increased metal dispersion, reduced reduction temperature, and lowered coke formation were contributed by a Ce promoter. According to the authors’ results, Ni-based catalysts enhanced the gasification process by increasing syngas production and reducing tar formation.

Tursun et al. studied decoupled triple-bed biomass gasification consisting of a biomass pyrolyzer, a tar reformer, and a char combustor. Olivine and NiO/olivine were used as catalysts for hydrogen-rich gas production. The results show that NiO/olivine was superior with a reduction of tar yield by 94%. When using the NiO/olivine catalyst, syngas with a H_2_ content of 56.1 vol % was obtained.^142^

Although catalysts have such desirable effects on gasification, they have a limited lifetime due to the products of side reactions and/or structural changes in the catalyst. Catalyst deactivation is caused by CH_4_ cracking and Boudouard reactions (the reaction of solid carbon with carbon dioxide to produce carbon monoxide^143^), causing coke deposition on the catalyst active sites. These detrimental effects lead to the deactivation of the catalyst and require it to be repaired or replaced by a new catalyst.^141,144^ Elbaba and Williams studied hydrogen production from waste tires through two-stage pyrolysis gasification.^145^ Ni/Al_2_O_3_ and Ni/dolomite were used as catalysts, and their deactivation was investigated over four cycles of use. Between the two catalysts, Ni/dolomite gave a higher experimental hydrogen yield and a higher theoretical hydrogen potential than the Ni/Al_2_O_3_ catalyst. Further, the lowest carbon deposition (2.8 wt %) was seen when using Ni/dolomite, but for the Ni/Al_2_O_3_ catalyst the carbon deposition was found to be 18.2 wt %. Reacted catalysts were subjected to detailed analysis by transmission electron microscopy to determine the existence of nickel, sulfur, and carbon in the reacted catalysts. According to their findings, the nickel of the Ni/Al_2_O_3_ catalysts was deactivated due to reactions with sulfur and carbon deposition.

Tar removal is essential for biomass gasification systems due to tar having hazardous ingredients. Tar removal methods are divided into two categories based on where the tar removal is carried out. These methods are termed in situ and ex situ or inside and outside of the gasifier itself, respectively.^146^ In ex situ catalysis, the tar is condensed and picked up for catalytic treatment outside of the gasifier when the gaseous tar gets converted. However, the catalyst layer is placed downstream of the reactor in in situ catalysis during conversion of gaseous tar. In situ catalysis is more effective owing to the conducting volatiles of the biomass pyrolysis, so it presents a high degree of tar removal^147,148^ due to direct interaction with volatiles and their arranged distribution.^149,150^ This classification of tar removal methods is also termed as primary methods (in situ) and secondary methods (ex situ) by some researchers.^151−153^

If tar elimination occurs outside of the reactor, it is appropriate for treating produced gas and can be categorized into three methods: (1) physical purification, (2) high-temperature thermal cracking, and (3) catalytic cracking.^146^ Wet and dry purifications are techniques that include filtration, aqueous or organic liquid scrubbing,^154^ absorbing, cyclone separation, etc. (used in physical purification).^155^ Even though physical purification methods are simpler, they have some drawbacks. For instance, these techniques require heating and cooling steps; they produce large amounts of liquid waste or wastewater when a scrubber is used; and the tar energy is not utilized.^146,156^ Thermal cracking is applied to raw gases that are obtained from gasification. When heated to a high temperature (>1000 °C), tar molecules are converted to lighter gases.^157,158^ In catalytic tar cracking, the tar molecules are cracked into lighter gases and soot when the raw gas is moved through a catalyst.^159^ To solve the above-mentioned tar problems, catalysts are used for tar cracking in which tar is converted to syngas, and the efficiency of the gasification is increased.^160^

There are different classifications of catalysts that are used for catalytic cracking purposes.^157^ Depending on their production method, catalysts can be divided into two groups: mineral and synthetic catalysts. Mineral catalysts include calcinated rocks (calcite, magnesite, and calcinated dolomite), olivine, clay minerals, and ferrous metal oxides, while synthetic catalysts include char catalysts, fluid catalytic cracking (i.e., zeolite) catalysts, alkali-metal-based catalysts, activated Al_2_O_3_, and transition-metal-based catalysts (Ni-, Pt-, Zr-, Rh-, Ru-, and Fe-based catalysts).^146,161,162^ Among these catalysts, natural minerals, alkali metals, transition metals, and noble-metal-based catalysts have been demonstrated to be notably effective for tar conversion and gas generation with good quality at comparatively low temperatures by many authors.^163−165^ Alkali metal (sodium, potassium, and calcium) and alkaline-earth metal catalysts are the most effective, followed by heavy metals.^166^ The Supporting Information of this study shows selected catalysts (Ce/Ni/Al_2_O_3_, Rh/Ce–Zr–O, and Fe/Ca_*x*_O) used for tar elimination and hydrogen production. In addition to the above, waste byproducts are also good alternatives to commercial or the previously mentioned catalysts. High reactibity, reusability, and cost effectiveness are desired properties of the catalysts. In view of these aspects, waste byproducts need to be considered as a source for catalysts.^128^ In the next section, apart from the above-mentioned catalysts, waste byproducts will also be considered in this review.

### 3.1 Alkali and Alkaline-earth Catalysts

Biochar contains naturally occurring alkali and alkaline-earth metals (AAEMs) and oxygen-containing functional groups that lead to the significant reduction or decomposition of tar.^167^ Inorganic portions of raw biomass or char consist of Ca, K, Mg, Al, P, and Si, and they can differ based on biomass type. Importantly, K, Ca, and Mg exist in high concentrations in biomass, and these inorganic elements affect gasification reactivity.^168,169^ Considering these points, alkali and alkaline-earth metal ingredients in biomass play an important role in biochar catalytic activity for tar reforming.^170^ Jiang et al. studied the catalytic effects of inherent AAEM species on biomass gasification.^171^ In order to evaluate the effect of inherent AAEMs, two different biomass materials (rice steam and rice husk) were demineralized by deionized water and a dilute HCl solution for comparison with untreated biomass feedstock. The experiments were carried out under steam at 900 °C. The results show that AAEMs improved the H_2_ and CO_2_ production while preventing the formation of CO, CH_4_, C_2_H_4_, and C_2_H_6_. Both heterogeneous and homogeneous hydrocarbons reforming and water-shift reactions were supported by inherent AAEMs. Additionally, AAEMs also had an important role in thermal cracking and tar reforming.

The porosity and surface area of biochar in addition to the inclusion of AAEM elements such as Na, K, and Ca cause biomass char to be reactive. Researchers have carried out CO_2_ gasification of pistachio nutshell char in a thermogravimetric analyzer (TGA) by applying alkali (Na, K), alkaline-earth (Ca, Mg), and transition metal (Fe) nitrates.^172^ The results showed that the catalytic effect on the enhancement of carbon conversion was achieved with Na-char and followed by Ca-char, Fe-char, K-char, Mg-char, and then raw char. In contrast to raw char, the existence of Na resulted in enhanced char reactivity by a factor of 2.36. Different Na catalyst loading was also investigated in a range of 3–7 wt %. The required time for carbon conversion is reduced (from 22 to 14 min) with increased loading from 3 to 5 wt %, and increasing the loading further had an adverse effect on catalyst activity.

K is one of the alkali metals that is used as a catalyst in gasification. It is considered the most active catalyst among the alkali metals. K accelerates the diffusion of the gasifying agent into carbon and thus leads to the formation of microstructures and thereby results in an increased reaction rate.^166^ Zhang et al. studied sorption-enhanced gasification of tobacco stalks by using steam as the gasification agent in a fixed-bed reactor.^173^ The effects of temperature, catalyst type, and catalyst loading were evaluated for hydrogen production. When using the selected catalysts of K_2_CO_3_, CH_3_COOK, and KCl, increasing the temperature from 600 to 700 °C and the loading of K_2_CO_3_ and CH_3_COOK enhanced the effects of the catalyst on the gasification of biomass for hydrogen production. On the contrary, increasing the loading of KCl resulted in a decrease in hydrogen yield and carbon conversion because of the inhibition of the gasification process. The maximum carbon conversion efficiency of 88.0% and hydrogen yield of 73.0% were achieved by applying 20 wt % of K loading in the K_2_CO_3_ precursor at 700 °C.

In order to improve syngas quantity and reduce the tar content, alkali and alkaline-earth catalysts are applied to algal biomass (*Cladophora glomerata L.*) through the steam gasification process.^174^ NaOH, KHCO_3_, Na_3_PO_4_, and MgO commercial catalysts were used in the process, of which NaOH was found to be superior for hydrogen production and also contributed to the conversion of char and tar decomposition. Increasing the temperature from 700 to 900 °C resulted in decreasing the tar content in the produced gas and increasing hydrogen yield.

Waste materials such as MSW and agricultural waste possess heavy metals, alkali metals, and alkaline-earth metals. In a study, the effects of constant concentrations (0.7 wt %) of Na, K, and Ca catalysts and S/B ratio on gasification efficiency for syngas production were investigated.^175^ Artificial waste composed of sawdust and polypropylene (PP) was gasified in a fluidized-bed gasifier with different S/B ratios (1.0–2.0). Increasing the S/B ratio from 1.0 to 1.5 increased the syngas production, while further enhancement to 2.0 caused adverse effects on the gasification process. According to the authors’ findings, either Na or K catalysts led to enhancement in the molar percentages of H_2_ and CO and decreased CH_4_ and CO_2_ content.

In contrast to the positive increase in hydrogen production, the inherent alkali metal content of biomass can be a problem at higher temperatures due to agglomeration of K and Na on bed material, which blocks the gasification process. Depending on biomass type and growth conditions, biomass ash includes inorganic materials such as silicon, calcium, potassium, and relatively small amounts of aluminum. These components can react with bed material, i.e., sand, and create a eutectic mixture through the combustion of biomass at high temperature. A eutectic mixture that has a low melting point of 754 °C covers the sand and forms bridges between sand particles.^176^ Rasmussen et al. studied the gasification of both straw and wood pellets in an allothermal fluidized-bed gasifier. In the study, different gasification temperatures in the range of 750 °C–950 °C were applied, and the agglomeration problem was observed at 950 °C when using straw as a feedstock. In contrast to straw, the same temperature was applied to the wood pellet feedstock, and no agglomeration was observed.^177^ Compared to the fixed-bed gasifier, the fluidized-bed gasifier offers great mixing between the biomass, gasifying agent, and gas–solid contact, but agglomeration is a crucial issue for biomass gasification in the fluidized-bed gasifier.^178−180^ The nature of the biomass may have an effect on agglomeration. K, Na, Si, and alkaline-earth metals that are inherently found in herbaceous plants contribute to ash formation. From those, K and Na are major elements that cause agglomeration.^181^ According to Nuutinen et al., bed materials containing silicon dioxide (SiO_2_) and K or Na cause the agglomeration.^182^ However, granule bed materials rich in Mg prevent agglomeration, thanks to minor components of potassium aluminosilicate, magnesium iron silicate, sodium aluminosilicate, sodium calcium aluminosilicate, and SiO_2_ particles. Thus, granule beds are more favorable for fuels that have a high alkali metal content.

### 3.2 Transition Metal Catalysts

A transition metal is defined as “a metal that forms one or more stable ions that have not totally used their d orbitals.”.^183^ Due to not filling of their d orbitals, transition metals are able to switch their oxidation states and give or take electrons from other molecules. Hence, tar decomposition is carried out with greater capacity due to the active element states.^184^ From the point of view of steam and dry reforming of methane and hydrocarbons, transition metals are considered good candidates for catalysts.^161^ Iron, cobalt, copper, nickel, and their compounds are widely used catalysts in the gasification process.^185^ Transition metal catalysts, can be used as hybrid catalysts due to the carrying properties of both heterogeneous and homogeneous catalysts. Transition metal nanoparticles have a high surface area and energy, ensuring they are active catalysts. However, the coking and sintering of relatively big metal particles adversely affects the total surface area and activity of these catalysts.^186^ On the other hand, Rh, Ru, Pd, Pt, etc. are termed as novel metal catalysts and have been used for reducing tar in the biomass gasification process. Although these catalysts are effective in dissociating tar into fuel gas, compared to nickel and conventional catalysts, their prices are very high.^187^

A different type of catalyst is used in SCWG to improve that process. For instance, alkali metals, metals, and metal oxides are widely used as catalysts in SCWG. Apart from these, rare metals such as Pt, Pd, and Rd are also employed in SCWG. However, these kinds of catalyst are useful at lower temperature and are not employed at higher temperatures due to decreasing catalytic activity.^188^ Doped metal oxides are the latest catalytic structure that can be applied for SCWG. Mastuli et al. employed SCWG to produce hydrogen using oil palm fronds as biomass feedstock.^189^ The authors developed a new catalyst structure that is composed of nanosized Zn-doped MgO catalysts with *x* values between 0.005 and 0.20 in Mg_1–*x*_Zn_*x*_O. The synthesized catalyst, which uses a self-propagating method, shows a decrease in surface area with increasing amount of Zn doping. However, the observed crystallite size was not more than 50 nm. According to the results, the highest H_2_ yield of 56.9% and lowest CO yield of 7.2% were achieved at Mg_0.80_Zn_0.20_O (*x* = 0.20). When compared with noncatalytic SCWG, H_2_ and CO content was increased by 438.1% and decreased by 82.4%, respectively.

Thanks to its high mechanical strength, olivine can be used as a primary catalyst to decrease tar content. Olivine ((Mg, Fe)_2_SiO_4_) includes natural iron oxides that strongly influence the olivine catalytic activity. Rapagnà et al. studied catalytic steam gasification of almond shells in a bubbling fluidized-bed gasifier by using 10 wt % of Fe/olivine catalyst synthesized via the impregnation method.^190^ The experiments were performed at temperatures between 800 and 830 °C, and blank (olivine) was used for comparison of the effect of a catalyst on gas and hydrogen yield. According to the results, a 10 wt % Fe/olivine catalyst exhibited superior stability and increased both gas and hydrogen yield by almost 40% and 88%, respectively. Furthermore, a 16% reduction of CH_4_ was achieved. Both reforming activity and reduction in tar concentration were realized by the Fe/olivine catalyst. When both economic and environmental causes are considered, the Fe/olivine catalyst is a good option for eliminating tar formation in the gasification process.

The gasification of biomass has sequential reaction stages that can be classified into (1) pyrolysis of biomass to generate char and (2) reaction of biomass char and the gasifier agent to produce a gaseous product. Among them, char gasification is the rate-limiting stage in the process. Jiao et al. studied the CO_2_ gasification of sawdust char with application of a K-modified transition metal composite.^191^ In the study, the effects of the gasification temperature, CO_2_ adsorption, and K-modified Co, Fe, Ni, and Ce metals were evaluated. Based on the results, the temperature is a crucial variable that influences char gasification. When the temperature is increased from 700 to 800 °C, carbon conversion increased 2.55 times in a 40 min reaction. Even at lower temperatures, the composite catalysts (KCo, KNi, KFe, and KCe, listed in order of catalytic performance) showed improvement in the char conversion. The results showed that both adsorbed quantity of CO_2_ and CO_2_ decomposition activity on the catalyst surface each affected the catalytic activity of the CO_2_ gasification.

The gasifier type also strongly affects catalytic performance. Hydrothermal gasification is a promising technology that converts high moisture content biomass to hydrogen-rich gas. Hydrothermal gasification has been applied to the distillery, oil refinery, and petrochemical complex waste streams.^192^ The catalytic activity of MnO_2_, CuO, and Co_3_O_4_ transition metal catalysts with different amounts of catalyst loading (20, 40, and 60 wt %), temperatures (300–375 °C), and reaction times (15, 30, and 45 min) was studied for hydrogen production. The results showed that distillery wastewater had the highest potential for hydrogen production among the considered waste streams. From the view of gasification efficiency and H_2_ mole fraction, the amount of catalytic activity of the various catalysts was found to be in the order of Co_3_O_4_, CuO, and then MnO_2_. At the operating conditions of 375 °C and 45 min reaction time, 40 wt % loading of Co_3_O_4_ was found to be appropriate for hydrogen production from distillery wastewater. Additionally, char formation was significantly reduced by using the catalyst.

Fe, Mg, Mn, Ce, Pt, Pd, and Ru are also used as dopants in Ni-based catalysts to enhance both the gas composition and the calorific value of the produced gas.^115^ Ni catalysts are attractive for enhancing hydrogen production. In the next section, Ni-based catalysts are discussed in more detail.

#### 3.2.1 Ni-Based Catalysts

Ni-based catalysts have recently attracted attention due to their unique properties such as supporting hydrogen and water–gas shift reactions,^189^ high activity, and lower cost.^193,194^ Nickel catalysts are regarded as the most efficient transition metal catalysts, allowing opportunities for tar cracking and reforming. Additionally, nickel catalysts also enhance the quality of the produced gases obtained from the gasification process.^195^ Many researchers have evaluated Ni-based catalysts for hydrogen production. However, in addition to the above-mentioned superior properties, coking and sulfur poisoning cause the deactivation of nickel active sites. These issues can be solved by either dispersing Ni nanoparticles on a support doped with an alkali metal or alloying with carbon.^196^

Peng et al. studied air-steam gasification of the wood residue using a research-scale fluidized bed. Two different types of metal catalysts (Ni/CeO_2_/Al_2_O_3_) at different catalyst loadings (20, 30, and 40%) were examined for catalytic activity. To investigate the effect of process parameters on the catalytic activity, different residence times (20, 40, and 60 min) and gasification temperatures (750, 825, and 900 °C) were examined. In parallel, noncatalytic experiments were also carried out to decide the optimal conditions that increase tar cracking and enriched hydrogen/syngas production. According to their results, the high temperature (900 °C) and high catalyst loading (40%) are suitable for tar cracking and enriching hydrogen/syngas production.^197^

The high metal surface area and high thermal stability are key features of Ni/Al_2_O_3_ catalysts. However, these kinds of catalysts generally face problems such as deactivation by coke deposition on the active sites and sintering. To overcome catalyst deactivation, the process configuration, optimization of process conditions, catalyst enhancement with different Ni loadings, additives, and supports have been investigated.^198−200^ Artetxe et al. studied eliminating the tar derived from biomass gasification via catalytic steam reforming on Ni/Al_2_O_3_ catalysts.^201^ Different Ni loadings (5%–40%) and varied tar model compounds (phenol, toluene, methyl naphthalene, indene, anisole, and furfural) were studied both individually and as a mixture at 700 °C with S/C ratio of 3 and 60 min on a gaseous stream. Based on the results, 20 wt % of Ni loading gave 90% of higher tar conversion and 63% of H_2_ potential. Among the tar compounds, anisole and furfural gave the highest conversion (75% and 68%, respectively) and H_2_ potential (45% and 43%, respectively), whereas methyl naphthalene showed the lowest activity.

The implementation of CaO into catalyst structures is good both for reinforcing the catalyst structure and as an in situ CO_2_ sorbent. For this reason, CaO has been widely applied in thermochemical conversion processes as a catalyst/sorbent and for tar-reforming purposes.^202,203^ Sisinni et al. investigated converting the topping atmosphere residue (a complex mixture of cyclic and polycyclic aromatic hydrocarbons) and CH_4_, to generate pure H_2_ by applying a CO_2_ sorbent.^204^ In the study, a hazelnut shell was used as the biomass material, and experiments were carried out in a fluidized-bed microreactor with steam. Commercial Ni catalysts and calcined dolomite (CaO/MgO) were used as catalyst precursors. The calcined dolomite that is used as a bed material behaves as both a reforming catalyst and CO_2_ sorbent. They found that both combinations of catalyst and sorbent were superior in moving away the topping atmosphere residue and CH_4_, and the conversion reached almost 100%. The sorption of CO_2_ promoted the water–gas shift increase, and H_2_ content in the syngas reached over 90%.

CaO is one of the tar-reforming agents that it is generally used because of its affordable cost and abundance.^205^ Calcined dolomite, calcined limestone, and calcined CaCO_3_ are some examples of materials including CaO.^206,207^ Li et al. studied corn stalk pyrolysis gasification by employing various calcium-based absorbents and NiO-based catalysts to produce H_2_.^208^ Based on the experimental results, calcined CaCO_3_, calcined limestone, and calcined dolomite were crucial to enhance the produced gas with regard to the concentration and yield of H_2_. This happened because of in situ CO_2_ absorption and the used types of CaO. Further enhancement of CO_2_ absorption and H_2_ production was achieved by calcined dolomite because of the inherent Mg species inside. Regarding the catalyst’s effect on the process, NiO/CaO bifunctional catalysts/absorbents were found to perform better than the calcined dolomite since its produced gas had lower H_2_ concentration, and the yields of H_2_, CO, and CO_2_ were higher. However, the concentration and yield of H_2_ were both improved when NiO/γ-Al_2_O_3_ catalysts along with calcined dolomite were used. The maximum H_2_ concentration of 85.1% and very little amount of CO_2_ were achieved with 15 wt % loading of NiO.

As mentioned earlier, SCWG has advantages when applied to wet or high moisture content biomass feedstock. The SCWG of wheat straw using Ni and other metal catalysts has been evaluated.^193^ The catalytic activity and the stability of Ni catalysts were evaluated through three stages. Ni, Fe, and Cu catalysts supported on MgO were prepared by the wet impregnation method. The prepared catalysts were used in SCWG inside a batch reactor at 723 K. The experimental results show that the order of the tested catalysts in terms of performance was Ni/MgO, Fe/MgO, and finally Cu/MgO. Then, various catalysts such as MgO, ZnO, Al_2_O_3_, and ZrO_2_ were examined as supports for the Ni. Based on the support materials, the order of catalytic activity of the Ni was Ni/MgO, Ni/ZnO, Ni/Al_2_O_3_, and Ni/ZrO_2_. The last stage included examining the effects of Ni loading and its hydrogen production effectiveness, and also the stability of Ni/MgO catalysts was investigated. The results showed that Ni catalysts exhibited critical deactivation in the SCWG process.

Even if Ni metal precursors are able to be modified by other transition metals (Fe, Co, Mn, and Cu) and noble metals (Pt, Ru, Pd, etc.), promoted with rare earth metals (La, Ce, and Pr), alkali (K, Na, Li, etc.), and alkaline-earth metals (Mg, Ca, Sr, and Ba), preparation methods and the Ni precursor are also highly important to improve the catalytic activity, stability, and resistance to coke formation and sintering.^209^ Therefore, the selection of an appropriate Ni precursor and preparation method is required to improve the catalytic performance. Recent research has mostly focused on enhancing the stability and activity of Ni catalysts by supporting with other catalysts, metal addition, and investigating the effects of Ni particle size. The impregnation method^187^ and sol–gel method^188^ are used for supporting nickel-based catalysts, where coimpregnation^189^ and coprecipitation^190^ are used for promoted nickel-based catalysts.

### 3.3 Carbon-Based Catalysts

Lignocellulosic biomass that consists of cellulose, hemicellulose, lignin, and lower amounts of inorganic minerals is used to produce biochar by thermochemical processes. Biochar has the potential to be used in various applications thanks to its unique properties such as large specific surface area (SSA), porous structure (micropore, mesopore, and macropore), functional groups, high reliability, low cost, and simple recovery of deactivation.^210−213^ One of the applications is using biochar as a solid catalyst to obtain biofuel and value-added chemicals from biomass.^214^ Biochar is generated as a byproduct of a gasification system that has important potential to be used in a wide range of applications. For instance, the low-temperature circulating fluidized bed gasifier in Denmark was designed to generate energy from biomass. Annually, this plant produces 64 tons of biochar residues that require use in sustainable applications.^215^

Not only is the char surface an essential aspect of tar reforming but also the porous structure of char is an important factor. Buentello-Montoya et al. investigated the porous structure of regular char obtained by pyrolysis and activated char that was activated physically using CO_2_ for tar reforming at temperatures between 650 and 850 °C.^216^ Their results show that higher tar conversion was achieved using activated char at 650 and 750 °C, while it presented more deactivation than the regular biochar. At a higher temperature (850 °C), two biochar catalysts exhibited the same performance, and tar (mixture of benzene, toluene, and naphthalene) removal efficiency reached 90% within the 3 h experiment duration. In contrast, the mesoporous and microporous chars exhibited higher initial tar conversion, but coking occurred due to rapid deactivation. The study proved that meso- and macroporous biochars are applicable alternatives for tar steam reforming. One of the advantages of char is its good catalytic activity due to its active sites, carbon structure, and alkali and alkaline-earth metal content. Furthermore, when many active metal oxides were loaded on char, it creates char-supported catalysts, and the catalytic activity of char is enhanced.^156^

Due to the abundance of feedstock, low cost, large surface areas, stability of both the acidic and basic areas, and the physical/chemical properties of biochar and ability to promote metal, biochar has become a widespread topic of study.^217−220^ However, each catalyst precursor has some disadvantages. For instance, Ni-based catalysts are very good at eliminating tar but can be deactivated during coking and tar conversion efficiency decreases. Owing to its unique properties, char and carbon-based catalysts have also attracted attention recently. Hu et al. investigated the catalytic activity of a gasified pine sawdust char promoted Ni catalyst and the effects of operation parameters on the produced gas composition.^221^ According to the results, among four different Ni loadings (2%, 4%, 6%, and 8%), char-promoted 6% Ni-loaded catalyst, 800 °C temperature, and a 0.5 s gas residence time gave optimal results. If the Ni content increases (0%–8%), the H_2_ content rises (25%–43%), but the CO content showed a slight decrease as well. It can be concluded from the study that char-promoted Ni catalysts can be used as a cheap catalyst.

The char catalytic performance is determined by factors including biomass precursor and origin, gasification parameters, catalytic condition, type of gasifier, and tar composition.^222,223^ Furthermore, the surface functional groups, surface area, and porous structure of char are also crucial for catalytic performance.^218^

Another key point to consider is the inhibiting effect of the inorganic content of char. There is some AAEM content, as the biomass precursor also includes elements such as Si, Al, and P that can have a negative impact on the gasification process and deactivate the char catalyst.^168^ The inhibiting effects of Al, Si, and P are demonstrated by some authors.^224,225^ One of the reasons for the inhibition is that melted ash formation covers the remaining char and prevents conversion of char at the next stage.^226^ Rizkiana et al. investigated the effect of varied biomass ash, namely, brown seaweed/BS, eel grass/EG, and rice straw/RS, as catalysts to enhance the gasification process. To determine the catalytic activity of biomass ash, low rank coal was used as a feedstock. BS and EG ash showed higher catalytic activity due to higher AAEM content. RS ash that contains high silica or silica-containing ashes created aggregation of ash particles on the coal surface, and this led to a decreased total active surface area, which results in the deterioration of its reactivity.^227^ Hence, it is required to evaluate the inorganic composition of biomass feedstock.

### 3.4 Natural Mineral Catalysts

Dolomites, CaMg(CO_3_)_2_, are natural minerals consisting of magnesium and calcium carbonates that can degrade into oxides at high temperatures. Dolomites and other naturally occurring catalysts may contain trace minerals like SiO_2_, Fe_2_O_3_, and Al_2_O_3_, of which iron oxide especially has an important role regarding catalytic activity.^228,229^ The hydrothermal gasification of walnut shells, hazelnut shells, and almond shells has been studied in a batch reactor using natural mineral salts as catalysts.^230^ Trona, dolomite, and borax were used as the natural mineral catalyst precursors. In the study, the temperature and pressure were varied across the ranges of 300–600 °C and 88–405 bar, respectively. Based on the results, trona was found to be the most effective catalyst with regard to the H_2_ yield (mol H_2_/kg C in the biomass) at 600 °C. The hydrogen yields of the hazelnut, walnut, and almond shells that were catalyzed in the presence of trona increased by 82.4%, 74.1%, and 42.4%, respectively. Additionally, hazelnut shells had a higher lignin content (40.0 wt %) than the other hard-nut shell precursors (walnut 35.4 wt %, almond shell 28.8 wt %) while using natural mineral catalysts, which was effective in degrading the lignin content of such hard-nut shells.

Olivine, (Mg, Fe)_2_SiO_4_, is a natural mineral catalyst consisting of magnesium oxide, iron oxide, and silica.^229^ Olivine can show good catalytic activity after calcination, and loading with active metals (i.e., Fe, Ni, Cu, Ce, etc.) can improve the catalytic performance of olivine. Meng et al. studied the gasification of pine sawdust in a circulating fluidized bed using a Ni–Fe bimetallic olivine-based catalyst. The catalyst was synthesized by the wet impregnation (WI) and thermal fusion (TF) method. Based on the results, tar reduction increased with the process temperature. Compared to a nonactive bed material (silica sand), a 40.6% reduction of tar content was attributed to raw olivine. Calcination of olivine further increases the catalytic activity of olivine catalysts. The presence of Fe_2_O_3_, NiO, and NiO-MgO in 1100-WI-olivine catalysts decreased the tar content by 81.5% compared to that obtained from the raw olivine catalyst. For 1400-TF-olivine, 82.9% of tar reduction was achieved as compared to that attained using raw olivine.^231^ A similar result was determined in the study of Rauch et al.^232^ They investigated the effects of calcination process on olivine catalyst, by using two different olivine precursors that have different iron contents. The results showed that the calcination process is important for oxidation of Fe ions to enhance tar reduction and increase the catalyst activity.^232^ Another study was carried out by Christodoulou et al. to exhibit the effect of calcination of olivine on tar reduction. They compared the performance of calcinated and uncalcinated olivine at the same conditions. The results showed that calcinated olivine yielded 5.7 g/Nm^3^ of tar content, while uncalcinated olivine yielded tar content of 9.5 g/Nm^3^ at 750 °C. Futhermore, increasing temperature up to 800 °C yielded lower tar content of uncalcinated olivine and calcinated olivine to 2.9 g/Nm^3^ and 1.9 g/Nm^3^, respectively.^233^

Feedstock species and the pretreatment method can also considerably affect tar yields. Torrefaction is one of the thermochemical methods that is carried out at temperatures of 200–320 °C. Torrefaction pretreatment decreases the moisture content, increases the energy density, and enhances the reactivity of the feedstock during gasification and combustion. Berrueco et al. evaluated the operating conditions of temperature and bed material on the yields and composition of gas as well as tar content from gasification.^234^ Norwegian spruce and Norwegian forest residues were used as the feedstock and were torrefied at 275 °C. Then, experiments were carried out in a fluidized-bed reactor using two different bed materials (sand and dolomite) at 0.5 MPa and at temperatures of 750 and 850 °C. Results showed that the dolomite catalyst increased tar reduction and increased the generation of gas components. Also, the temperature increase contributed to both the cracking reactions and the tar reduction.

Oyster shells can be recovered, recycled, and then used in many applications from acting as an adsorbent material to an antibacterial material. One of the applications of recycled oyster shells is as a catalyst in gasification. Cheng and colleagues investigated the catalytic gasification of automobile shredder residue (ASR) that has trace pollutants including volatile sulfur and chlorine to produce hydrogen gas.^235^ In this study, oyster shells with high calcium contents were used as catalysts for increasing hydrogen production. The authors applied a 900 °C temperature and varied the catalyst addition (5%–15%) in a fixed-bed and a fluidized-bed gasifier. The results showed that higher ASR decomposition and maximum H_2_ and CO yields of 12.12% and 10.59%, respectively, were achieved in the fluidized-bed gasifier.

### 3.5 Catalyst Alternatives to Waste Byproducts

Sustainability is crucial for both the environment and economy, so using waste products is a good method to increase the sustainability of many processes. One application of waste materials is using them as catalysts. Red mud, aluminum dross, fly ash, slag from iron manufacturing, sludge, chicken eggshells, marine shells, snail shells, coconut shells, rice husks, and gold mine waste all have elements that enable their use as catalysts.^236^ Although this kind of waste material suffers from properties like impurities, lower surface area, etc., it is a good option for minimizing waste and reducing environmental impacts.^236^ On the contrary, Ni, Pt, Pd, Rh, and Ru possess the highest catalytic activity, long-duration stability, and low carbon deposition through tar cracking, but they are rare in nature and not economically sustainable.^237^

Eggshell is a good source of CaO due to its high CaCO_3_ content. The shells decompose under high temperatures and produce CaO.^238,239^ Raheem et al. studied the catalytic gasification of lipid-extracted microalgae biomass to produce hydrogen-rich syngas.^240^ Eggshell was used in the catalytic gasification as a source of CaO, and different loadings were applied (10, 30, and 50%). By increasing catalyst loading, the H_2_ yield was increased, but the CO and CO_2_ yields decreased. As mentioned previously, CaO can contribute to CO_2_ capture thanks to the adsorption ability of CaO.

Even though nickel and noble metal catalysts have superior properties in tar cracking, they are expensive and can also produce toxic byproducts. To solve these problems, natural materials must be considered as alternative catalysts. Oyster and mussel waste shells are calcium-rich materials that can be used for tar removal instead of calcined dolomite and olivine, as they do not need to be mined and have less adverse effects on the environment. Oyster and mussel waste shells have been employed to synthesize nanomaterials to produce clean syngas from MSW.^237^ Both catalysts similarly increased gas yields at 800 °C. The oyster-derived catalysts exhibited a higher tar removal at higher temperature (1000 °C) because of their effect on syngas yield, and they decreased the soot yield by improving the quality of syngas. Additionally, the PAH concentration was decreased by using the oyster-derived catalysts, and the H_2_/CO ratio was increased by almost 2.8 times. In summary, oyster-derived catalysts perform better than mussel-derived catalysts, as the oyster-derived catalysts have two times higher SSA and bigger crystallite size than the mussel-derived catalysts.

Marble has a calcite nature, and its processing creates a large amount of waste marble powder (WMP) as a byproduct. Irfan et al. carried out a novel study on utilizing WMP as a catalyst in the MSW gasification process.^241^ The experiments were carried out in a laboratory-scale batch-type fixed-bed reactor to investigate the effect of utilizing WMP on the CO_2_ sorption, steam-reforming capability, and char gasification while using steam as a gasifying agent. Different temperatures, steam flow rates, and WMP-to-MSW ratios were evaluated from 700 to 900 °C and at 2.5–10 mL/min, and 0, 0.25, 0.5, 0.75, and 1 were evaluated, respectively. The results showed that increasing the WMP-to-MSW ratio leads to increased H_2_ production. However, CO_2_ adsorption is decreased under the same conditions.

Using wastes as biomass feedstock is a good method to both produce high value-added products and minimize wastes that would otherwise be disposed of in a landfill and create environmental problems. Cement kiln dust is generated from Portland cement processing, and it is generally stored in a landfill to meet environmental regulations. Hamad et al. investigated various gasification parameters on biomass feedstocks (cotton stalks, rice straw, and corn stalks) to produce hydrogen-rich gas.^242^ The chosen parameters were the oxygen-to-fuel ER (0.12–0.4), reaction temperature (700–850 °C), reaction duration (45–120 min), and catalyst species. The chosen catalysts included marly clay, calcium hydroxide, dolomite, and cement kiln dust. Based on the results, the best performance is achieved for an ER of 0.25 and 90 min reaction duration at 800 °C for calcined cement kiln dust and CaO. Among the catalysts, calcium hydroxide resulted in a higher concentration of H_2_ (45%) and CO (33%) for the gasification of cotton stalks. Using calcined cement kiln dust for gasification of the same material not only yielded relatively higher concentrations of H_2_ (39%) and CO (33%) but also gave a higher overall gas yield of 1.5 m^3^/kg with cement kiln dust compared to other agriculture residues of corn stalks (1.3 m^3^/kg) and rice straw (1.03 m^3^/kg).

In Table 3, catalyst type was summarized in the view of their representatives, characteristics, advantages/disadvantages, and target products.

**Table 3:** Catalysts in the View of Their Representatives, Characteristics, Advantages/Disadvantages, and Target Products

type	representatives	characteristics	advantages/disadvantages	target products	ref
AAEMs	K (K_2_CO_3_ and KOH)	increased reaction rate	Advantages:	hydrogen and syngas	(166, 243−246)
			high mobility		
		inherently found in biomass	creating micropore structure on carbon feed	reducing tar and soot ingredients	
			increased reaction rate		
			Disadvantages:		
			volatility of potassium species (i.e., KCl)		
			deactivation of potassium		
			agglomeration at higher temperatures above 800 °C		
			difficulty in catalyst recovery		
transition metal catalysts	Ni	high activity	Advantages:	reducing tar content	(195, 247, 248)
			low cost compared to other transition metal precursors		
			Disadvantages:	enhancing the quality of gaseous product	
			deactivation caused by sintering and carbon formation		
			scarce sources such as Pt, Ru, Rh, Ir, and Pd		
carbon-based catalysts	biochar, activated char	large specific surface area (SSA)	Advantages:	tar conversion	(210, 212, 213, 222, 249)
			high reliability		
		porous structure	low cost		
			simple recovery upon deactivation		
		functional groups	good catalytic activity		
		good catalytic activity	Disadvantages:		
			requires modification for use as the support		
			declining active sites over time		
natural mineral catalysts	dolomite	relatively favorable catalytic activity	Advantages:	increasing the quality of gaseous product	(128, 194, 243, 250)
			low cost		
			abundance	providing 95% and more tar reduction	
			Disadvantages:		
			require further cleaning process for accessing the active component of the material		
			decrease in the mechanical strength with time		
catalyst alternatives to waste byproducts	material that has CaCO_3_ content such as egg shell, oyster shells, etc.	abundance	Advantages:	increasing H_2_ yield	(228, 229, 251)
		high CaCO_3_ content	low cost		
			minimizing waste product	promising CO_2_ absorption	
			Disadvantages:		
			deactivation due to particle agglomeration		
			require modification of the active site		

As seen in Table 3, each type of catalyst has advantages and disadvantages. Until now, steps have been taken to eliminate the disadvantages of catalysts, and further research is going on. It is known that catalysts are used in thermochemical systems including gasification for producing biofuels, heat, power, etc. Academic researchers have been developing and finding new catalysts which are active, effective, and cost efficient. However, the other crucial thing is whether they are used in industrial plants or not. Hence, STEEP analysis of catalysts is carried out to achieve future sustainable development with the aid of the past and current situation of catalysts. In the next section, STEEP analysis is deeply evaluated.

## 4 STEEP Analysis of Catalysts

To enhance global welfare in light of social, environmental, and economic sustainability, sustainable development goals were proposed by the United Nations in 2015.^252^ Green chemistry and cleaner production methods are concepts that include new techniques and practices to help prevent adverse environmental effects. In order to reduce CO_2_ emissions and other potential pollution, adopting green chemistry principles is important for sustaining our future. Green chemistry can be defined as preventing waste and pollution through employment of materials, processes, or practices. These processes and practices include preventing pollution, decreasing consumption of chemical products that have negative impacts on both human health and the environment, eliminating hazardous content from existing products and processes, designing chemical products, and processes that have less structural hazards.^253^ These terms also extend to the reduction and efficient usage of hazardous/nonhazardous materials, energy, water, and other natural resources.^254,255^ Cleaner production contributes to sustainable development through the effective management of both resources and energy and the improvement of technology, which also helps the political side and all stakeholders in the industry.^256^ Catalysis is an important area in the chemical sector that plays a crucial role in many fields such as energy conversion, materials synthesis, environmental protection, and human health.^257^

**Figure 2:**
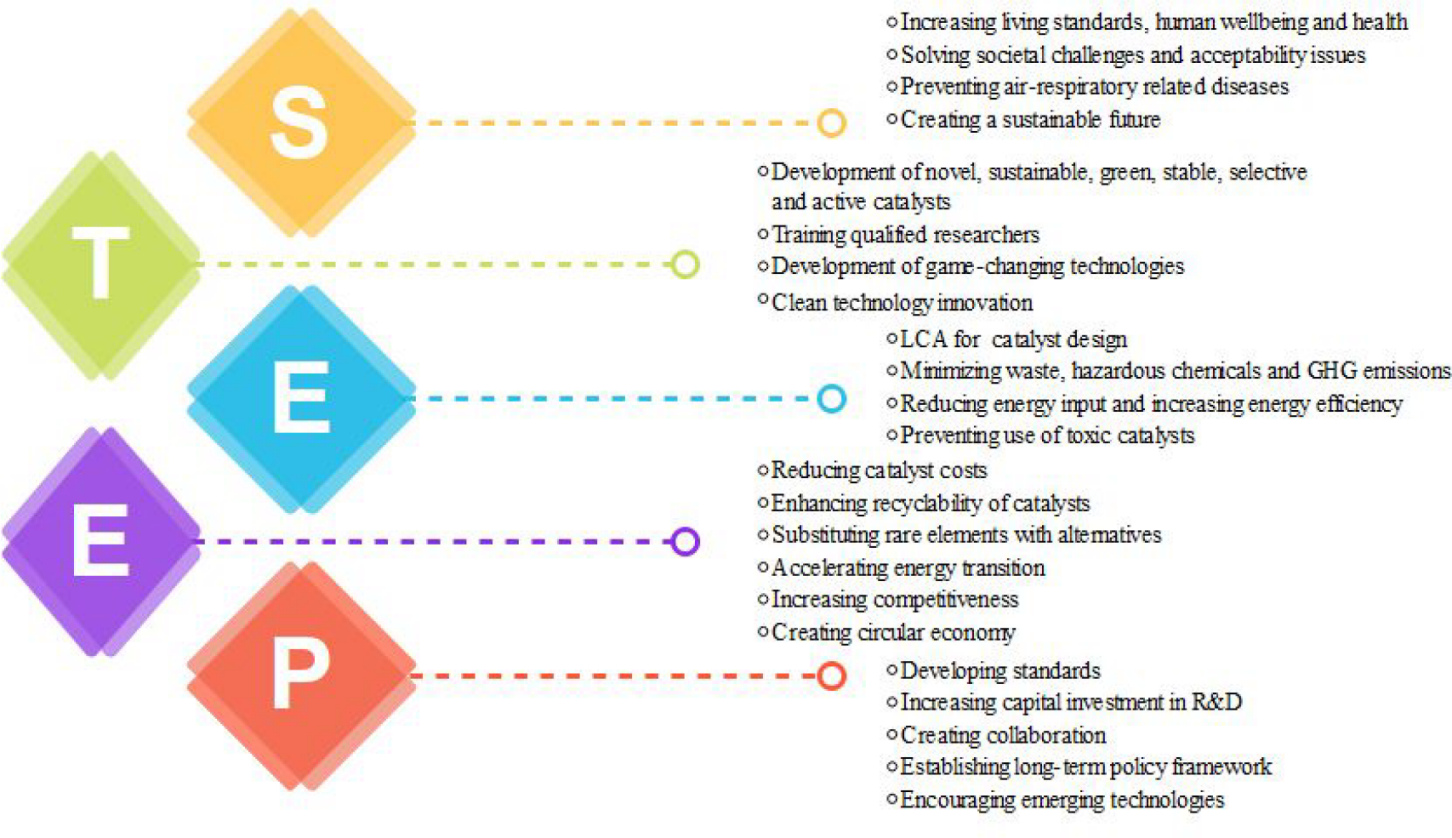
Social, technological, environmental, economic, and political (STEEP) analysis of catalysts (derived with permission from refs (115, 252, and 256−270)).

In this review, STEEP analysis was evaluated for catalysts (shown in Figure 2) with five dimensions that are social, technological, environmental, economic, and political. STEEP analysis is a powerful tool for evaluating all aspects considered in the decision-making process of a system or service, in both academic and industrial fields. It is a decision-making tool for understanding sustainability. The STEEP analysis was used to analyze the past and current state of the catalyst that was applied in gasification. In this regard, technical reports, roadmaps and academic essays were reviewed in the view of all related aspects.

Hydrogen is not just an energy carrier but also a feedstock for many industrial and energy-related applications. The chemical sector is the largest industrial consumer of both oil and gas, and it is responsible for 30% of the energy usage among all industrial sectors.^264^ Approximately 20%–30% of the world GDP is impacted by catalysis and catalytic processes. At present, 30 of the 50 chemicals with the largest volume are produced using catalysts. Every year, 20 billion tons of CO_2_ are emitted into the atmosphere due to these 50 highest volume processes.^261^ Catalysts plays a key role in the chemical industry and seriously affect both today’s and future environmental conditions. Catalysts can produce greener products, more sustainably and more efficiently. Hence, they contribute to the reduction of CO_2_ emissions and prevent future energy difficulties.^269^

In order to understand catalyst consumption through gasification processes, the IEA Bioenergy database regarding “Gasification of Biomass and Waste” was examined.^271^ Evaluation was carried out excluding power, heat, combined power, and heat as outputs. Also, nonoperational status plants were excluded. Table 4 shows the projects that were evaluated under these criteria.

**Table 4:** Biomass and Waste Gasification Projects^a^

owner	name	technology	product	TRL	catalyst	location
Advanced Biofuels Solutions, Ltd.	Swindon Advanced Biofuels Plant	fuel synthesis	SNG, hydrogen	8	N.A.I.	Swindon, United Kingdom
Cutec	Synthesis Cutec Clausthal-Zellerfeld	fuel Synthesis	FT liquids	4–5	N.A.I.	Clausthal-Zellerfeld, Germany
bDillinger Saar GmbH	Project Selma	plasma gasification	hydrogen	9	-	Premnitz, Germany
ECN	MILENA Gasifier	indirect gasification (MILENA-technology)	clean syngas	4–5	olivine (bed material)	Petten, Netherlands
Enerkem	Varennes Carbon Recycling	fuel synthesis	biofuel and renewable chemicals	6–7	N.A.I.	Varennes, Canada
Enerkem	Synthesis Enerkem Sherbrooke	fuel synthesis	SNG, cellulosic ethanol, methanol	4–5	N.A.I.	Sherbrooke Canada
Enerkem	Westbury commercial demonstration facility	fuel synthesis	chemical-grade syngas, methanol, ethanol, and other chemicals	6–7	N.A.I.	Westbury, Canada
Enerkem Alberta Biofuels LP	Edmonton Waste-to-Biofuels Projects	fuel synthesis	chemical-grade syngas, methanol, ethanol, and other chemicals	8	N.A.I.	Edmonton, Canada
Neue Energy Premnitz	Premnitz Project	plasma gasification	hydrogen	9	high-temperature and low-temperature pellet catalysts are used at the end of the process in the water-shift reactor	Premnitz, Germany
NREL	Thermochemical User Facility (TCUF)	different technologies including gasification, etc.	various chemicals	4–5	no catalyst	Golden, United States
RWE Power AG	MFC within ITZ-CC	other gasification technologies	clean syngas	4–5	no Catalyst	Bergheim-Niederaussem, Germany
TUBITAK	TRIJEN	FT liquids	biofuel	4–5	N.A.I.	Kocaeli, Turkey
Uni Stuttgart	Magnus 200 kW pilot plant for SEG	fuel synthesis	clean syngas	4–5	N.A.I.	Stuttgart, Germany
Xylowatt, University Catholic of Louvain-la-Neuve (UCL)	Test Gasifier Plant TGP	other gasification technologies	syngas	4–5	N.A.I.	Louvain-la-Neuve, Belgium

a FT: Fischer–Tropsch. TRL: Technology Readiness Level. SNG: Synthetic natural gas. N.A.I.: No available information.

b Project Selma is currently planned, so there is information about its operation and operation conditions.

Hydrogen is an enormous fuel for various chemicals and fuels. It can be seen in Table 4 that syngas and hydrogen are output products of some projects, while certain projects aim to produce diversified outputs including biofuels, chemicals, ethanol, methanol, etc. Most of these technologies are in TRL 4–5 and still require reaching the commercialization level (TRL 8–9). Heat, power, combined heat, and power plants have TRL 8–9, which plays an active role in the commercial area. Unfortunately, the projects including fuel synthesis and the production of hydrogen, syngas, methanol, etc. are still at demonstration levels that require scale up in order to be used for commercial purposes.

Biomass gasification enables the production of synthetic gas, and when combined further with FT or the purifying process, biofuel- or hydrogen-rich syngas is produced, respectively.^272^ Natural mineral catalysts, alkali and alkaline earth metal catalysts, Ni-based catalysts, and zeolites are commonly used catalysts in the gasification process to enhance the H_2_ content of syngas. However, most projects in Table 4 use another way to reduce tar content. Tar reduction is carried out in the projects either in their reactor with novel reactor design or in the additional gas cleaning and gas upgrading units. Additionally, this kind of unit brings additional cost to the system that is an obstacle to the commercialization of biomass gasification. Instead of this, it is necessary to develop a new catalyst that is more active and stable against carbon formation and sintering.^269^ Regarding catalyst usage in the systems, not much information from IEA Bioenergy “Gasification of Biomass and Waste” could be attained. Further research was carried out about patents held by companies. Although there is no information on exactly which catalyst is used in the facilities, a zinc oxide bed is used for catalytic reforming purposes to remove residual tar and convert the molecular weight hydrocarbons into H_2_ and CO in one of the patents of Enerkem Inc.^273^ Also, Enerhem Inc. holds patents about the catalyst used for producing hydrogen and synthesis gas. To give an illustration, the company developed catalysts consisting of nickel and/or cobalt supported on a support that includes a mixed oxide containing metals, such as aluminum, zirconium, lanthanum, magnesium, cerium, calcium, and yttrium. Developed catalysts are effective to convert carbon dioxide and methane to carbon monoxide and hydrogen, respectively.^274^ To identify catalysts that are used for producing hydrogen with a gasification process, an extensive patent search was made on the Espacenet database using the terms “catalyst”, “gasification”, and “hydrogen production”. Nickel is a cheap catalyst precursor, although it must be upgraded in view of its catalytic activity, hydrogen selectivity, and deactivation. To overcome its negative aspects, a hydrogen production catalyst and preparation method were developed and patented by Yancheng Fuhua Environmental Prot Industry Development Co. Ltd. The nickel precursor was combined with other elements and components including the zinc ion, magnesium ion, ammonia, ethyl orthosilicate, and manganese ion to improve its catalytic activity.^275^ JP2006068723A,^276^ CN103263923A,^277^ JP2006122841A,^278^ and FR2809030A1^279^ are some nickel composite catalysts that are combined with carriers such as porous carbon, zeolite, and other elements. Combining nickel with other catalyst precursors is not a new topic of research, and it has been studied for several years.

Technological development includes many aspects that affect the improvement and promotion of technology. Training qualified researchers has a place in both the social and technological aspects of the STEEP analysis. From the societal perspective, qualified people will change the thought process, while from the technological aspect, a trained and qualified researcher will contribute to the existing technology and knowledge related to their field. Additionally, training qualified researchers will contribute to enhancing R&D activity.^267,280^ The deactivation of catalysts is one of the major obstacles for this field, and it requires intensive scrutinization and study. Poisoning, fouling, thermal degradation, vapor compound formation, vapor–solid or solid–solid reactions, attrition, and crushing are all reasons for catalyst deactivation.^281^ Catalysts have a limited lifetime, and used catalysts can be either recycled, downcycled, or discarded. Although disposing of the catalyst is more attractive due to being the most cost-effective option, it can create negative environmental impacts due to its composition.^282^ Therefore, from a sustainability point of view, practical, efficient, reliable, and economic catalyst regeneration methods need to be developed. For catalysts used in the catalytic conversion of biomass, there are some unique challenges: (i) the requirement of a stable catalyst, (ii) more selective reactions, and (iii) integrating catalysis with separation.^283^ In order for the research and development activities carried out to be put into action, it is necessary to bring a cost estimate. Hence, the NREL (National Renewable Energy Laboratory) has developed a tool for accelerating catalyst development, which is namely CatCost, that combines cost estimation methods and resources in an intuitive tool suite.^284^ Reducing the cost of catalysts and the required investments will contribute to wider adoption of the technology and will increase its competitiveness in both the industry and energy sectors. Beyond developing catalysts with desired properties, the catalyst industry will have a positive impact on both social and economic aspects. The social aspect will also be affected in this situation by increased job opportunities and improved living standards and human health. Because of that, the tool of The Jobs and Economic Development Impact (JEDI) model has been developed by NREL to forecast the economic impacts of foundation and operation plants for both the local and state levels.^285^ With the aid of this tool, the number of jobs and economic impacts to a local area can be estimated..

Fossil-fuel-based energy and chemical production are cheaper than renewable resources as they comprise a more mature technology. However, GHG emissions are tightly coupled with what resources are employed for which purpose; i.e., fossil-fuel-based energy and chemical production release more GHG than renewables ones.^265^ To overcome this problem, economists and policymakers should accelerate the energy transition from fossil fuels to renewable resources for both the energy and chemical transformation industries.^268^ Biomass can both reduce GHG emissions and meet environmental policies and determined reduction targets, while also improving the chemical production and making use of renewable energy in local carbon resources such as CO_2_ and waste. Additionally, by increasing the capital investment in R&D of new catalysts, establishing collaborations between industry and academia by policymakers, public–private partnerships will accelerate reaching sustainability goals and transitioning to future energy forms.

Biomass is abundant, cheap, and carbon-neutral, enabling its use in different thermochemical processes to make high-value-added products. Although the gasification of biomass has many advantages as mentioned above, there are some disadvantages with regard to feedstock impurities, seasonal availability, and tar formation. Compared to other hydrogen production methods, the gasification process has a H_2_ generation efficiency in the range of 30%–40% with relatively lower production costs (1.77–2.05 $/kg) compared to other methods, especially electrolysis (10.30 $/kg).^286^ However, the efficiency of biomass gasification still needs to be improved to compete with other H_2_ production methods. To sum up, it can be concluded from STEEP analysis that catalysts have a crucial role in our life and economic development. Thus, specific focus on the development of special and effective catalysts for the gasification process is required.

## 5 Conclusion and Future Work

This study assessed hydrogen production via catalytic biomass gasification and performed the STEEP analysis of catalysts used in a wide range of vital processes in several industries and a circular economy.

Fuel and chemical industries are the most important sectors that use hydrogen. In both of these sectors, CO_2_ emissions are quite high due to using fossil-fuel-based sources. Apart from playing a critical role in energy applications, H_2_ also has an important role in the chemical industry, especially in the production of ammonia and methanol. The use of catalysts in the production of these resources by various thermochemical methods has an essential role as catalysts increase the energy efficiency, the rate of rectification, and the production efficiency. Biomass gasification is one of the thermochemical methods that produces green hydrogen as it utilizes carbon-neutral biomass feedstock. Catalysts are the key player in many processes including biomass gasification, FT synthesis, etc. As a priority in biomass gasification, alkali and alkaline earth metal catalysts, Ni-based catalysts, natural mineral catalysts, and waste byproducts are applied as catalyst precursors. However, each of these catalysts has merit and demerit to be improved to enhance the process and syngas yield. Through STEEP analysis, it was concluded that academic study on catalyst development and commercial applications of them do not progress simultaneously. Correspondingly, the STEEP analysis showed that catalyst development specifically for biomass gasification is required and needs to be applied by industry. In order to achieve a greener and more sustainable future, the currently used catalysts must move toward being more stable, efficient, economic, and reusable and must contribute to less energy-intensive processes.

## Supporting Information Available

Metal-based catalyst, Ni, dolamite, Rh, Fe, and H_2_ yield, and tar yield (PDF)

## Abbreviations List

SCWG: Supercritical water gasification
AES: Alkaline electrolysis systems
ER: Equivalence ratio
S/B: Steam-to-biomass ratios
S/C: Steam-to-carbon ratios
HTC: Hydrothermal carbonization
PAHs: Polycyclic aromatic hydrocarbons
AAEMs: Alkali and alkaline-earth metals
TGA: Thermogravimetric analyzer
PP: Polypropylene
SSA: Specific surface area
FT: Fischer–Tropsch
TRL: Technology readiness level
SNG: Synthetic natural gas
LCA: Life-cycle assessments
GHG: Greenhouse gases
R&D: Research and development
NREL: National Renewable Energy Laboratory
JEDI: The Jobs and Economic Development Impact
