# Supplementary material for: Review on Catalytic Biomass Gasification for Hydrogen Production as a Sustainable Energy Form and Social, Technological, Economic, Environmental, and Political Analysis of Catalysts
Source: ACS Omega. 2022 Jul 12;7(29):24918–41. doi: 10.1021/acsomega.2c01538 (PMC9330121; doi:10.1021/acsomega.2c01538)
Supplement: Supplementary file 1 — ao2c01538_si_001.pdf [file ao2c01538_si_001.pdf]

Supporting Information

A review on catalytic biomass gasification for hydrogen production as a sustainable energy form and STEEP analysis of catalysts

Fikret Muge Alptekin[a,b], Melih Soner Celiktas[a*]

f.mugealptekin@gmail.com, *soner.celiktas@ege.edu.tr

[a]Solar Energy Institute, Ege University. 35100 Bornova-Izmir. Turkey

[b]Robert M. Kerr Food and Agricultural Products Center, Oklahoma State University, Stillwater, OK, USA

0000-0003-2980-5403, 0000-0003-0597-5133

Table-S1. Effect of catalyst type and operating parameters on tar reduction and hydrogen production

Catalysts	Operation conditions	Tar model and inlet tar concentration (g/Nm^3)	Outlet gas composition (NmL/min) or (mol%)	H_2 yield %	Type of reduced tar g/Nm^3), and tar yield (%)	Ref.
Metal-based catalyst<br>Pt/MNC (MgO, NiO, SiO2)	-Bench scale unit consisting of a quartz reactor<br>-Temperature (680 °C and 750 °C)	Acetic acid, 40 g/Nm^3	H_2 (126.6), CO_2 (51.2), CO (40.3), and<br>CH_4 (0.1)	-	100% acetic acid	[1]
Ni/dolomite/ La_2O_3	-Fixed bed reactor<br>-Temperature (500°C–900°C)<br>-S/C molar ratio (0.5–2.5)<br>-Gas hourly space velocity (GHSV) (8,000–22,000 h^{-1})	Phenol (15%), toluene (50%), naphthalene (30%), and pryne (5%)	H_2, CO, CO_2, and a small amount of CH_4 (<0.3 mol%)	81% of H_2 at 750°C, S/B = 1, and GHSV of 14.000 $h^{-1.}$	N/A	[2]

$Ce/Ni/Al_2O_3$	-Oat hull pellet<br>-Two-stage fixed bed reactor<br>-Steam gasification agents<br>-Temperature (650°C –850°C)<br>-S/B ratio of 0.25–0.50<br>-GHSV of 2200 h^{-1}	N/A	H_2 (39.5%), CO (20.3%), CO_2 (21.5%), CH_4 (14.6%), and C_2 (2.16)	The highest yield of 39.5% H_2	Lowest tar yield of 2.1% at 650°C and S/B = 0.50	[3]
Rh/Ce-Zr-O<br>Ce-Zr-O, ZrO_2, CeO_2,<br>Rh/ZrO_2,<br>Rh/SiO_2,<br>Rh/CeO_2<br>Rh/CeO_2/Si O2,<br>Rh/ZrO_2/Si O2, and<br>$Rh/CeO_2/ZrO_2$	-Reaction temperature (575°C –730°C)<br>-Support chemical composition<br>-Rh loading percent (0.5% and 1.5%)<br>-Feed concentration of phenol and water<br>-Synthesis method (sol-gel, wet impregnation)	Phenol	H_2, CO, and CO_2	11.1 mol% of H_2 was achieved by Rh–35% ZrO_2/SiO_2	87% conversion of phenol was achieved by Rh–35% CeO_2/SiO_2 at 615 °C	[4]
Fe/Ca_xO	-Wood sawdust pellet<br>-Two-stage fixed bed reactor<br>-Different Ca/Fe molar ratios (2/1, 3/1, 4/1, 5/1)	Aromatics, phenol, naphthalene, acids, ketones, and furans	H_2, CO, CH_4, and CO_2	H_2 yield of 37.48% was obtained at Ca/Fe = 2/ 1.	Aromatics, furanes, acids, and ketones were removed by Ca/Fe = 2/1 ratio of catalyst	[5]
Ni/HZSM-5	-Corncob used as feedstock<br>-Two-stage fixed bed reactor<br>-Varied Ni loading % (6, 9, 13, and 16)<br>-Different temperatures (550°C–750°C)	Toluene	H_2, CO, and CO_2	The highest H_2 yield of 52.8 mmol/g was obtained by 9Ni/FZ5 catalyst.	88% tar conversion	[6]
Biochar	-Different biomasses: land	N/A	H_2, CO, CO_2, CH_4, C_2H_n,	49.3% of H_2 content	94.6% of tar conversion	[7]

	(corn stalk, Cs), coastal zone (reeds, Re), and marine biomass (*S. horneri,* Sh*)* were used.<br>-Bench-scale downstream combined fixed bed system was used.<br>-Different catalyst thicknesses (10 mm, 30 mm, 50 mm)<br>-Different temperatures (700, 750, 800, 850, 900°C)		and C_3H_n	was achieved by Sh-derived biochar catalyst<br>.	by Re-char catalyst	
Biomass char, calcined dolomite, olivine, FFC catalyst, biomass ash and commercial nickel catalyst, commercial biomass char (C.B. char)	-Fixed bed tubular reactor<br>-Different temperatures (700°C–900°C)	Naphthalene<br>Phenol	H_2, CH_4, CO, and CO_2	The highest H_2 content of 1.60 vol% was achieved at 900 °C.	−91% and 100% of phenol were converted by a Ni catalyst at 700 and 900°C, respectively<br>−99.6% and 100% of naphthalene was converted by C.B char and Ni+sand, respectively	[8]

N/A: Not available

References

(1) Cavalli, A.; Tetteroo, R.; Graziadio, M.; Aravind, P. V. Catalytic Reforming of Acetic Acid as Main Primary Tar Compound from Biomass Updraft Gasifiers: Screening of Suitable Catalysts and Operating Conditions. *Biomass and Bioenergy* **2021**, *146*, 105982. https://doi.org/10.1016/j.biombioe.2021.105982.

(2) Tan, R. S.; Tuan Abdullah, T. A.; Ripin, A.; Ahmad, A.; Md Isa, K. Hydrogen-Rich Gas Production by Steam Reforming of Gasified Biomass Tar over Ni/Dolomite/La2O3 Catalyst. *J. Environ. Chem. Eng.* **2019**, *7*, 103490. https://doi.org/10.1016/j.jece.2019.103490.

(3) Abedi, A.; Dalai, A. K. Steam Gasification of Oat Hull Pellets over Ni-Based Catalysts: Syngas Yield and Tar Reduction. *Fuel* **2019**, *254*, 115585. https://doi.org/10.1016/j.fuel.2019.05.168.

(4) Polychronopoulou, K.; Costa, C. N.; Efstathiou, A. M. The Steam Reforming of Phenol Reaction over Supported-Rh Catalysts. *Appl. Catal. A Gen.* **2004**, *272*, 37-52. https://doi.org/10.1016/j.apcata.2004.05.002.

(5) Zhang, X.; Yang, S.; Xie, X.; Chen, L.; Sun, L.; Zhao, B.; Si, H. Stoichiometric Synthesis of Fe/CaxO Catalysts from Tailored Layered Double Hydroxide Precursors for Syngas Production and Tar Removal in Biomass Gasification. *J. Anal. Appl. Pyrolysis* **2016**, *120*, 371-378. https://doi.org/10.1016/j.jaap.2016.06.005.

(6) Tang, W.; Cao, J. P.; Yang, F. L.; Feng, X. B.; Ren, J.; Wang, J. X.; Zhao, X. Y.; Zhao, M.; Cui, X.; Wei, X. Y. Highly Active and Stable HF Acid Modified HZSM-5 Supported Ni Catalysts for Steam Reforming of Toluene and Biomass Pyrolysis Tar. *Energy Convers. Manag.* **2020**, *212*, 112799. https://doi.org/10.1016/j.enconman.2020.112799.

(7) Li, J.; Liu, Z.; Tian, Y.; Zhu, Y.; Qin, S.; Qiao, Y. Catalytic Conversion of Gaseous Tars Using Land, Coastal and Marine Biomass-Derived Char Catalysts in a Bench-Scale Downstream Combined Fixed Bed System. *Bioresour. Technol.* **2020**, *304*, 122735. https://doi.org/10.1016/j.biortech.2020.122735.

(8) Abu El-Rub, Z.; Bramer, E. A.; Brem, G. Experimental Comparison of Biomass Chars with Other Catalysts for Tar Reduction. *Fuel* **2008**, *87*, 2243-2252. https://doi.org/10.1016/j.fuel.2008.01.004.
